# Supplementary material for: Study on the Effect of Pharmaceutical Excipient PEG400 on the Pharmacokinetics of Baicalin in Cells Based on MRP2, MRP3, and BCRP Efflux Transporters
Source: Pharmaceutics. 2024 May 29;16(6):731. doi: 10.3390/pharmaceutics16060731 (PMC11206988; doi:10.3390/pharmaceutics16060731)
Supplement: Supplementary file 1 [file pharmaceutics-16-00731-s001.zip › pharmaceutics-3001409-supplementary.pdf]

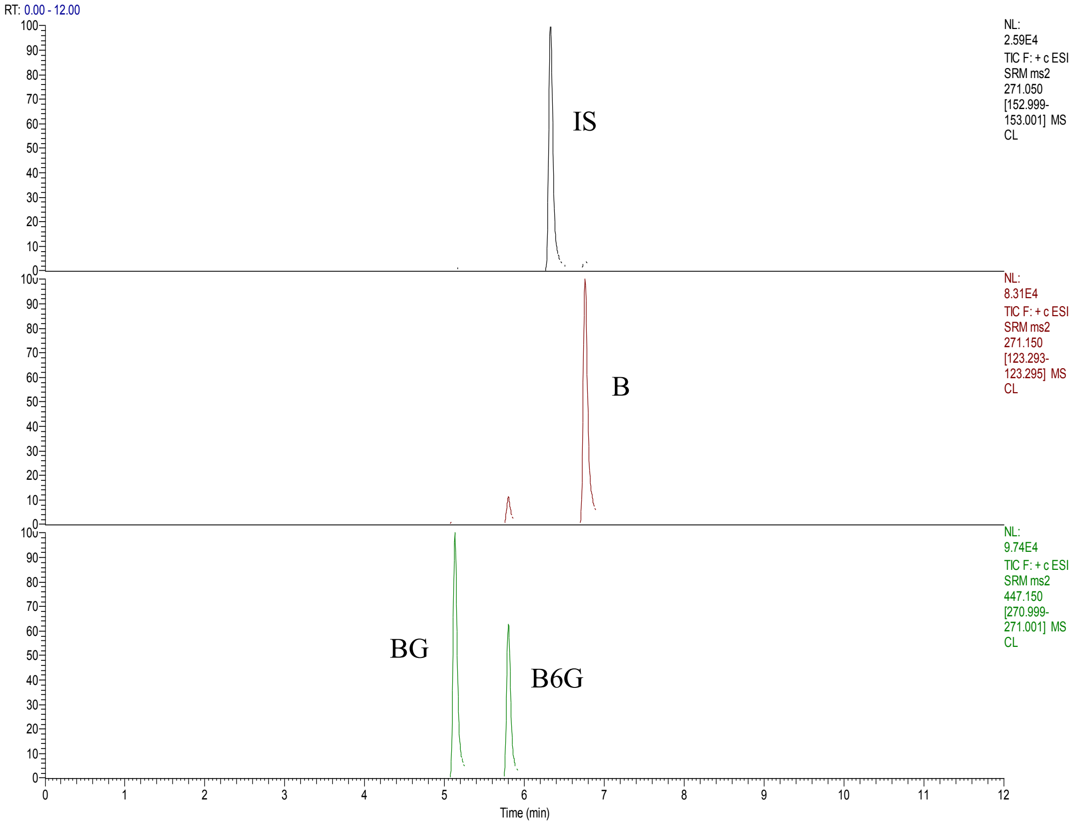

Figure S1. The SRM chromatogram of BG, B, B6G in cell lysate.

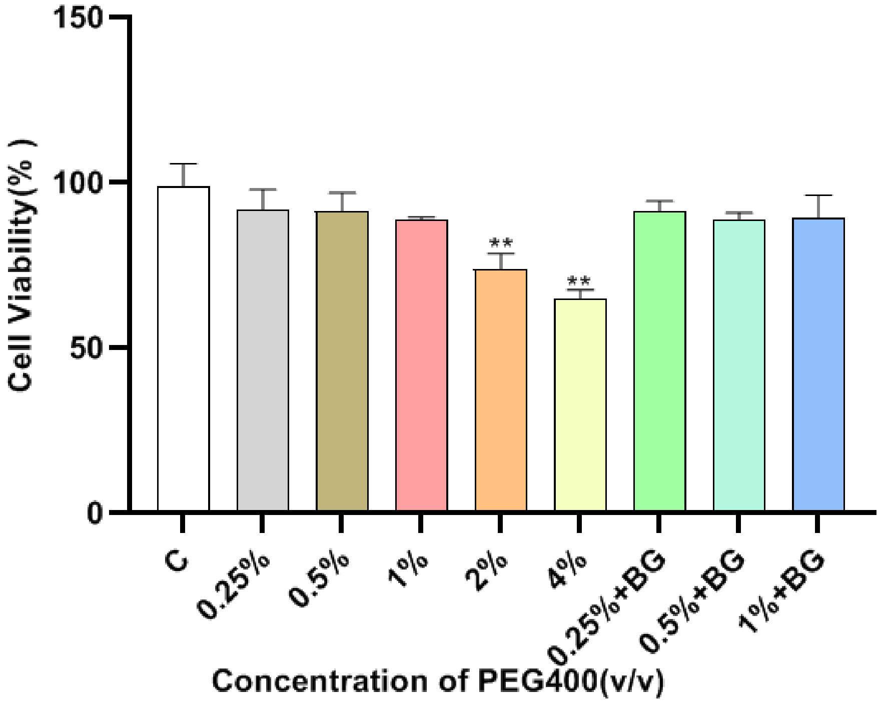

Figure S2. The effect of different concentrations of PEG400 on the survival of HepG2 cells. Compared to the control group, **** $p < 0.01$ **($\bar{x} \pm s$, $n=5$ **).**
